# The synergistic tumor growth-inhibitory effect of probiotic *Lactobacillus* on transgenic mouse model of pancreatic cancer treated with gemcitabine

**DOI:** 10.1038/s41598-020-77322-5

**Published:** 2020-11-23

**Authors:** Shan-Ming Chen, Wee-Wei Chieng, Szu-Wei Huang, Li-Jin Hsu, Ming-Shiou Jan

**Affiliations:** 1 Department of Pediatrics, Chung Shan Medical University Hospital, Taichung, Taiwan; 2 Department of Pediatrics, School of Medicine, Chung Shan Medical University, Taichung, Taiwan; 3 Institute of Biochemistry, Microbiology and Immunology, Chung Shan Medical University, Taichung, Taiwan; 4 Department of Post-Baccalaureate Veterinary Medicine, Asia University, Taichung, Taiwan; 5 Department of Medical Laboratory Science and Technology, Medical College, National Cheng Kung University, Tainan, Taiwan; 6 Institute of Medicine, Medical College, Chung Shan Medical University, 110, Sec 1, Jianguo N Rd, Taichung, 40246 Taiwan; 7 Immunology Research Center, Chung Shan Medical University, Taichung, Taiwan; 8 Division of Allergy, Immunology and Rheumatology, Department of Internal Medicine, Chung Shan Medical University, Taichung, Taiwan

**Keywords:** Cancer, Microbiology, Gastroenterology

## Abstract

Pancreatic cancer is one of the most lethal and chemo-resistant cancers worldwide. Growing evidence supports the theory that the gut microbiota plays an essential role in modulating the host response to anti-cancer therapy. The present study aimed to explore the effect of probiotics as an adjuvant during chemotherapy for pancreatic cancer. An *LSL-Kras*^*G12D/−*^-Pdx-1-Cre mouse model of pancreatic ductal adenocarcinoma (PDAC) was created to study the effects of using four-week multi-strain probiotics (*Lactobacillus paracasei* GMNL-133 and *Lactobacillus reuteri* GMNL-89) as an adjuvant therapy for controlling cancer progression. At 12 weeks of age, pancreatitis was induced in the mice by two intraperitoneal injection with caerulein (25 μg/kg 2 days apart). Over the next 4 weeks the mice were treated with intraperitoneal injections of gemcitabine in combination with the oral administration of probiotics. The pancreas was then harvested for analysis. Following caerulein treatment, the pancreases of the *LSL-Kras*^*G12D/−*^-Pdx-1-Cre transgenic mice exhibited more extensive pancreatic intraepithelial neoplasia (PanIN) formation. Combined treatment with gemcitabine and probiotics revealed a lower grade of PanIN formation and a decrease in the expression of vimentin and Ki-67. Mice that received gemcitabine in combination with probiotics had lower aspartate aminotransferase (AST) and alanine aminotransferase (ALT) levels. Notably, the use of high-dose probiotics alone without gemcitabine also had an inhibitory effect on PanIN changes and serum liver enzyme elevation. These findings suggest that probiotics are able to make standard chemotherapy more effective and could help improve the patient’s tolerance of chemotherapy.

## Introduction

According to GLOBOCAN 2018, pancreatic cancer is the 12th most commonly occurring malignancy (2.5%) and the 7th most lethal cancer (4.5%) worldwide. It was estimated that there were approximately 458,918 newly diagnosed cases of pancreatic cancer in 2018 leading to approximately 432,242 deaths^1^. The mortality rate is nearly identical to the incidence rate because patients seldom exhibit specific symptoms until late stages of the disease. Current literature shows that it still has one of the lowest 5-year survival rates of all cancers (< 10%)^2^. At present, the exact causes of pancreatic cancer are not well understood. However, some predisposing factors have been identified, such as smoking, obesity, diabetes, alcohol abuse, dietary factors, ethnicity, family history and genetic factors^3^. *Kras* oncogenic mutations at codons 12, 13, 61 are observed in > 90% of cases, and have been known as a driver of cancer development^4^. Inactivating mutations in tumor suppressor genes, including *TP53, CDKN2A,* and *SMAD4*, are also often detected in patients with advanced pancreatic cancer^2,4^.

Pancreatic ductal adenocarcinoma (PDAC), accounts for over 90% of patients with pancreatic cancer and is one of the most chemo-resistant and radio-resistant cancers^5^. Pancreatic intraepithelial neoplasia (PanIN) is well known as one of the major precursor lesions of PDAC. PanINs are noninvasive epithelial proliferations and metaplasia of the smaller pancreatic ducts (< 5 mm in diameter), and high-grade PanIN lesions are usually found simultaneously adjacent to PDAC^6^. Epithelial to mesenchymal transition (EMT), the phenotypic transition from epithelial cells to a mesenchymal cell state that facilitates cell migration, is considered a contributing factor to cancer metastasis and resistance^7^. Moreover, inflammation has been described as being associated with different stages of cancer development^7^.

Different *Kras*-*driven* genetically engineered mouse models (GEMMs) of PDAC which were developed by expression of the exogenous *Kras*^*G12D*^ oncogene and activation of a Pdx1-Cre transgene, have helped our understanding of carcinogenesis and therapeutic responses^8^. Previous studies have shown the anti-proliferative or proapoptotic properties of probiotics in human cancer cells/cell lines, such as gastric cancer cells and colonic cancer cells^9^. A recent study has shown that the intestine and pancreas microbiome is distinct and stage-specific in PDAC in mice and humans, and that the endogenous microbiome drives disease progression via induction of intra-tumoral immune suppression. It was suggested that microbial targeted therapies may reduce the risk of pre-invasive disease and may be of benefit as an adjuvant therapy to standard available therapies^10^.

The aim of the present study was to explore the effects of probiotic-based regimens when combined with chemotherapy drugs, to determine whether they can improve the side effects of medication during anticancer chemotherapy treatment.

## Methods

### Chemicals

Caerulein (Sigma-Aldrich, C9026), a cholecystokinin analogue derived from the Australian tree frog (*Litoria caerulea*), has been widely used to successfully induce pancreatitis in rodents^11^. Gemcitabine (2′, 2′-difluorodeoxycytidine) (Gemzar; Eli Lilly and Company, Indianapolis, IN, USA) is a fluorinated nucleoside analog that is converted to gemcitabine triphosphate in cells and inhibits DNA synthesis through its incorporation in DNA^12^. It is one of the main chemotherapy drugs used for the treatment of pancreatic cancer and as an adjuvant treatment after cancer resection^13^.

### Ethical statement

All animal use procedures were approved by the Institutional Animal Care and Use Committee of Chung Shan Medical University (approval no. 1897) and were performed in accordance with the Guideline for the Care and Use of Laboratory Animals (NRC, 2011).

### Genetically engineered transgenic mice

*LSL-Kras*^*G12D/-*^ (K) and pancreatic and duodenal homeobox (Pdx)-1-Cre (C) mice strains were obtained from the National Cancer Institute Mouse Repository in the USA. K and C mice were crossbred to obtain *LSL-Kras*^*G12D/−*^-Pdx-1-Cre (KC) transgenic mice. These strains were reared under specific pathogen-free conditions, and properly backcrossed to achieve a pure C57BL/6 genetic background, which was expanded at the National Laboratory Animal Centre, Taiwan, and later distributed to the Chung Shan Medical University Laboratory Animal Research Centre for further study. Mice of all genotypes were housed in a mouse room between 20–23 °C with a 12/12 h light-dim cycle. Laboratory Autoclavable Rodent Diet 5010 (Purina) and reverse osmosis purified water were provided at all times after the autoclaving process. At 12-weeks of age, KC transgenic mice were randomized to the treatment groups (gemcitabine and gemcitabine + probiotics), with no gender-related differences in the measured parameters.

### Pancreatitis induction and chemotherapy

In 12-week-old KC transgenic mice, pancreatitis was induced by 7 hourly intraperitoneal injections in the right lower quadrant with reduced dose caerulein (25 μg/kg dissolved in PBS 100 μl). This was then repeated 2 days later^11^. The KC transgenic mice were then randomly divided into four groups and treated with different dosages of gemcitabine (20, 50, 100 or 200 mg/kg). The initial gemcitabine injection was given intraperitoneally on the third day of the experiment, which was the day after the second injection cycle of caerulein treatment. Mice were injected every 3 days for a total of 10 injections.

### Probiotic oral gavage

*Lactobacillus paracasei* GMNL-133 and *Lactobacillus reuteri* GMNL-89 were provided by GenMont Biotech Inc., Tainan, Taiwan. There were two different doses of probiotics for mice, either GMNL-133: GMNL-89 in a 1:1/10 ratio (1.64 × 10^7^ CFU/0.02 kg: 1.64 × 10^6^ CFU/0.02 kg), or a 1:1 ratio (1.64 × 10^7^ CFU/0.02 kg: 1.64 × 10^7^ CFU/0.02 kg). Lyophilized live probiotic powder (1.64 × 10^7^ CFU/0.02 kg) was resuspended in sterile Milli-Q water and mixed well by vortexing. This was then freshly administered to the mice (0.2 ml) by oral gavage through a stainless-steel feeding tube 5 days per week for four weeks^12–14^.

### Blood biochemistry and histopathology

For the histological studies, the animals were sacrificed 30 days after the beginning of treatment. After anesthetization, the mice were disinfected on their ventral side with 75% ethanol^15^. Body weight was recorded for each mouse before sacrifice and pancreas weight was recorded right after they were sacrificed. To study the biochemical effects of caerulein, gemcitabine and probiotics on blood cells and biochemistry, the blood was collected after sacrifice by cardiac puncture and immediately sent to Axel Biotechnology Inc., Taiwan for blood cell counting and biochemical analysis. Pancreas tissue was aseptically removed from the mice, and a small piece of tissue was fixed with 10% neutral buffered formalin (TONYAR Biotech. Inc. Taiwan) for 24–48 h at 4 °C. The fixed tissues were trimmed to an appropriate size, embedded in paraffin, sectioned and then stained with Hematoxylin and Eosin (H&E)^16,17^. Vimentin (Vimentin (D21H3) XP Rabbit mAb, Cell Signaling Tech. # 5741) and Ki67 (Anti-Ki67 antibody KO tested, Abcam; ab15580) expression were detected using immunohistochemical staining methods in mouse pancreatic tissue. Immunohistochemical staining was performed using the UltraVision Quanto Detection System kit (Thermo Fisher Scientific Inc., Fremont, CA, USA) according to the manufacturer’s instructions^18^.

### Statistical analysis

Statistical software SPSS version 10.1.3C (SPSS Inc., Chicago, IL, USA) for Windows was used for recording data and analyzing results. Student’s *t-*test was used for the comparison of two means. Fisher’s exact test for categorical data was used as appropriate. A *P-*value < 0.05 was considered to indicate a statistically significant difference.

## Results

### Creation of pancreatic cancer animal models

**Figure 1:**
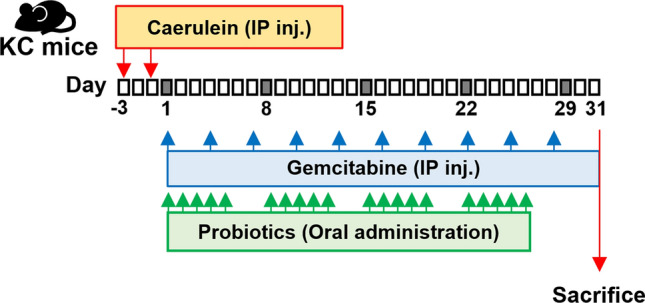
Schematic representation showing the time course for developing a mouse model of pancreatic cancer.

**Figure 2:**
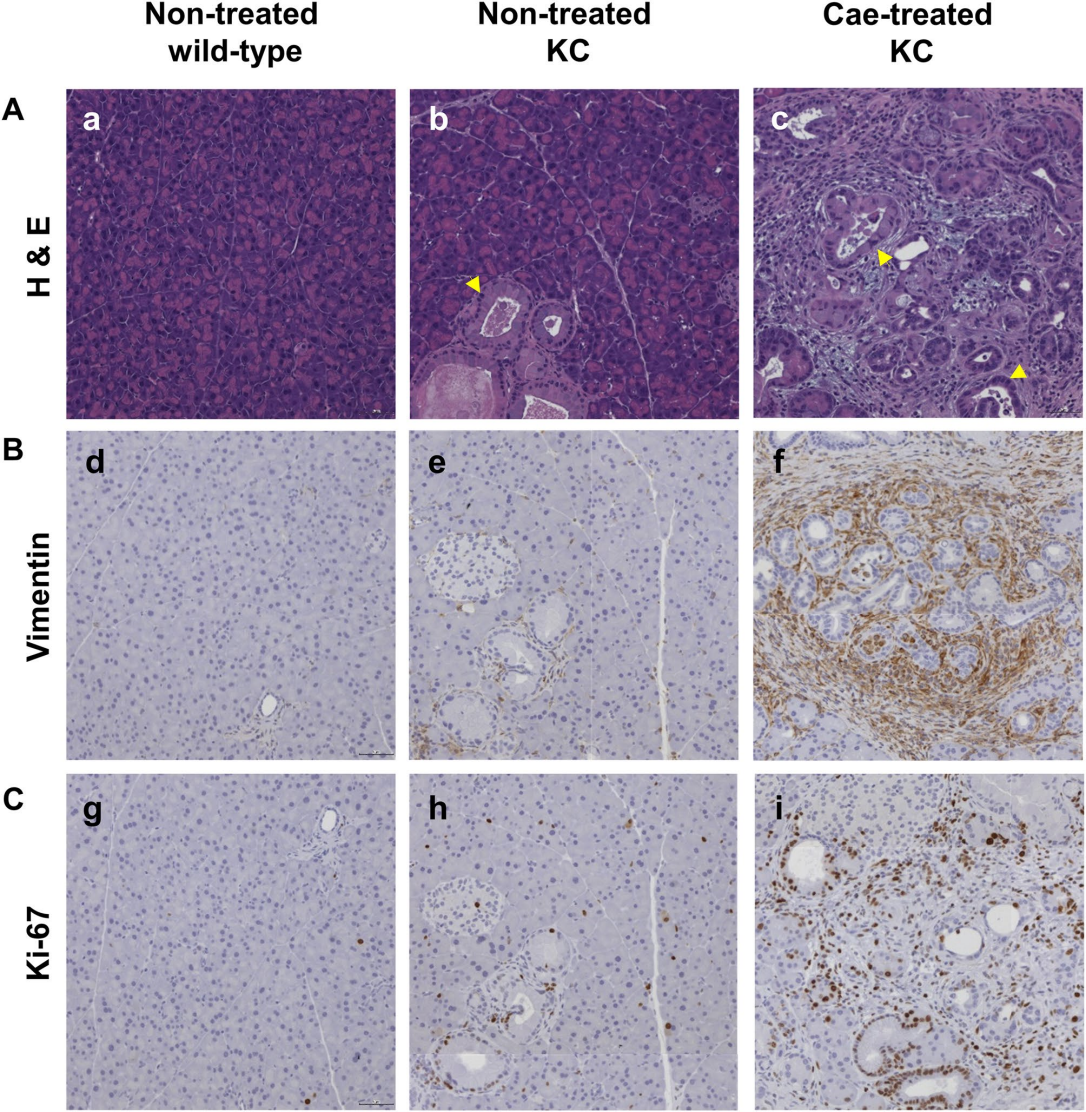
General pathological characteristics of PanIN lesions in a genetically engineered mouse model. Different staining images of pancreas histology at 10 × magnification in non-treated wild-type mice (n = 3), non-treated *LSL-Kras*^*G12D*^; PDX-1-Cre (KC) transgenic mice (n = 4), and caerulein-treated KC transgenic mice (n = 5). (**A**) H&E stained section of the pancreas shows architectural and cytological changes of the smaller duct, including tall columnar cells (arrow), papillary formations, variation in nuclear size (pleomorphism), and nuclear hyperchromasia in KC transgenic mice (b, c). (**B**) Vimentin is mainly expressed around the pancreas duct and the surrounding area in KC transgenic mice (e, f). (**C**) Increasing Ki-67 positive acinar cell number is observed in KC transgenic mice (h, i). Cae, caerulein; H&E, Hematoxylin and eosin; KC*, LSL-Kras*^*G12D/-*^; PDX-1-Cre transgenic mice; PanIN, pancreatic intraepithelial neoplasia.

The creation of pancreatic cancer animal models and the treatment process are shown in Fig. 1. Pancreas histology comparisons of non-treated wild-type mice (n = 3), non-treated KC transgenic mice (n = 4), and caerulein-treated KC transgenic mice (n = 5) are shown in Fig. 2. H&E staining of pancreas sections revealed a reactive duct with enlarged nuclei (arrowhead) in KC transgenic mice (Fig. 2b,c). Vimentin-positive stains around the pancreas duct (arrow) and its surroundings were significantly found in non-treated KC and caerulein-treated mice (Fig. 2e,f) in comparison to the non-treated wild-type mice. Ki-67 staining was also distributed in the pancreas of non-treated KC and caerulein-treated KC mice (Fig. 2h,i). Histological evidence indicates that the caerulein induced KC transgenic mouse model appropriately imitates PanIN.

### Effect of different dosages of gemcitabine

**Figure 3:**
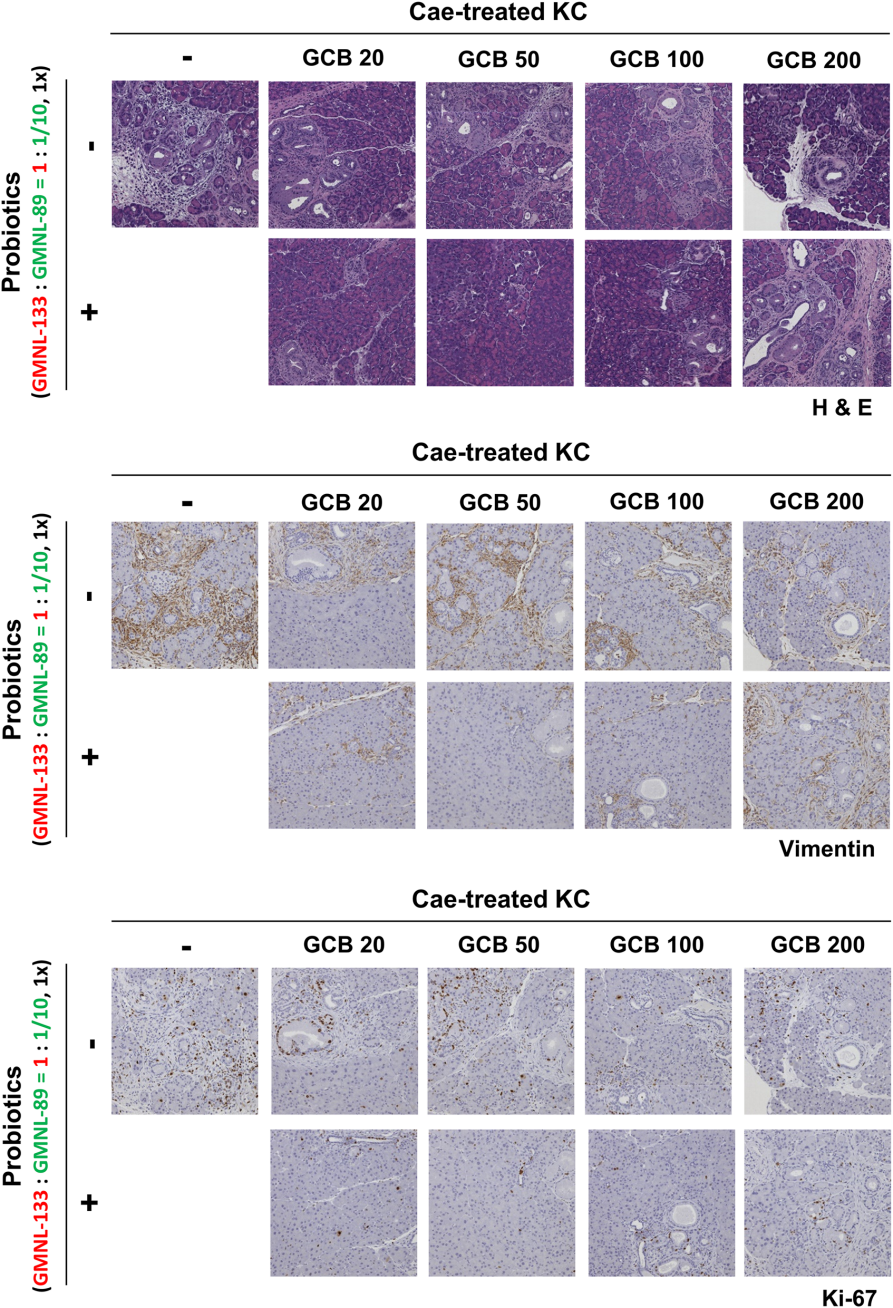
Representative images of pancreatic sections after various doses of gemcitabine in caerulein-treated KC transgenic mice with the co-administration of probiotics (GMNL133: GMNL89 in 1:1/10 ratio). The dosage of gemcitabine was 20 mg/kg (n = 3), 50 mg/kg (n = 3), 100 mg/kg (n = 3), and 200 mg/kg (n = 4). The combined application of gemcitabine and probiotics resulted in a lower grading of PanIN lesions by H&E staining and lower expression of vimentin and Ki-67. Cae, caerulein; GCB, gemcitabine; H&E, Hematoxylin and eosin; KC, *LSL-Kras*^*G12D/-*^; PDX-1-Cre transgenic mice; PanIN, pancreatic intraepithelial neoplasia.

Figure 3 shows the pancreas histology of KC transgenic mice treated with caerulein combined with different doses of gemcitabine (20, 50, 100, or 200 mg/kg). H&E staining of the pancreatic sections revealed that caerulein-treated KC transgenic mice who also received gemcitabine developed a lower grading of PanIN lesions, compared with those who received caerulein alone. A lower expression of EMT was also shown in vimentin staining, and a lower Ki-67 expression in Ki-67 staining as well (Fig. 3). The 50 mg/kg and 100 mg/kg gemcitabine treated groups showed superior outcomes based on their histological responses.

### Administration of dual-strain probiotics

**Figure 4:**
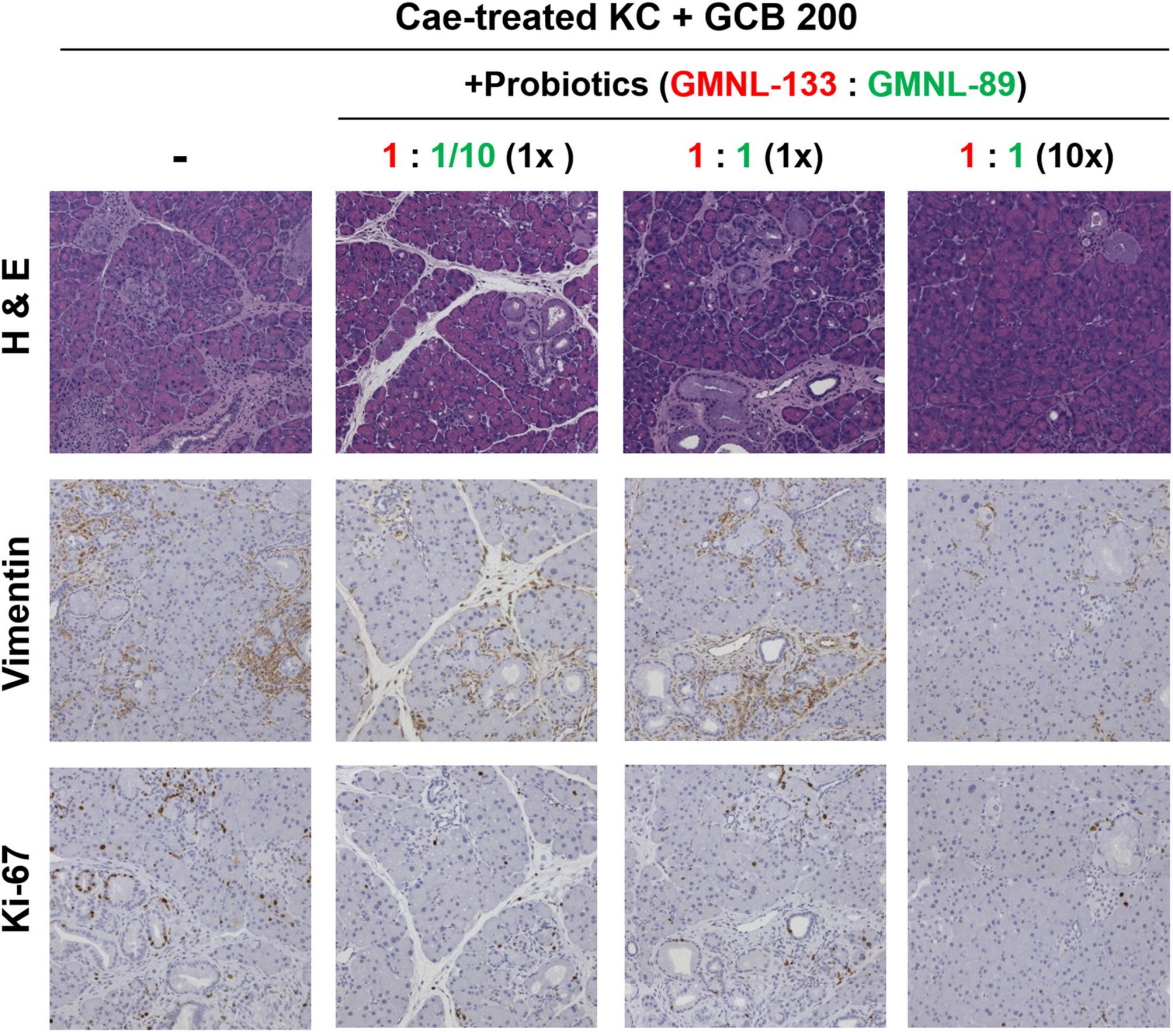
Representative images of pancreatic sections following a combination of various proportions and doses of probiotics with gemcitabine 200 mg/kg in caerulein-treated KC transgenic mice. No marked PanIN improvement was observed in the group that received the probiotics GMNL-133: GMNL-89 in a 1:1 ratio (n = 5) in comparison with the group that received GMNL-133: GMNL-89 in a 1:1/10 ratio (n = 4). A 10 × higher probiotic dose (n = 6) showed the lowest grade of PanIN progression in H&E staining and had the lowest expression of vimentin and Ki-67. Cae, caerulein; GCB, gemcitabine; H&E, Hematoxylin and eosin; KC*, LSL-Kras*^*G12D/-*^; PDX-1-Cre transgenic mice; PanIN, pancreatic intraepithelial neoplasia.

**Figure 5:**
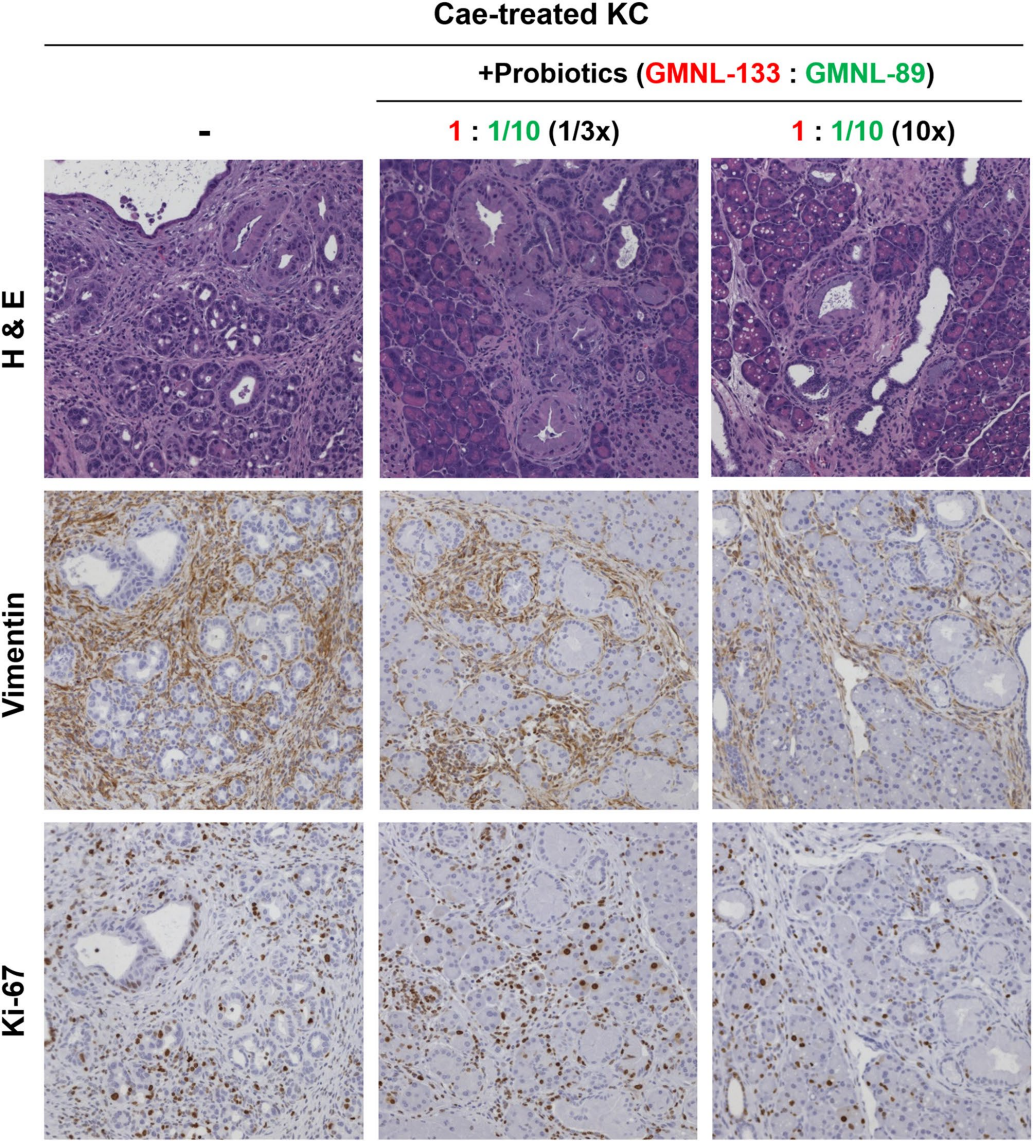
Representative images of pancreatic sections after receiving high-dose (n = 3) or low-dose (n = 3) probiotic treatment alone in caerulein-treated KC transgenic mice (GMNL133:GMNL89 = 1:1/10, 1/3 × vs. GMNL133: GMNL89 = 1:1/10, 10x). The group receiving 10 × higher probiotic doses showed a lower grade of PanIN progression in H&E staining and a lower expression of vimentin and Ki-67 staining. Cae, caerulein; H&E, Hematoxylin and eosin; KC*, LSL-Kras*^*G12D/-*^; PDX-1-Cre transgenic mice; PanIN, pancreatic intraepithelial neoplasia.

After caerulein treatment, the probiotics were administered by oral gavage and combined with gemcitabine at a dosage of 20 mg/kg (n = 3), 50 mg/kg (n = 3), 100 mg/kg (n = 3) or 200 mg/kg (n = 4). The group treated with gemcitabine and probiotics (GMNL-133: GMNL-89 in a 1:1/10 ratio) showed a lower grade of PanIN lesions in the H&E staining of pancreatic sections compared with those mice only receiving gemcitabine treatment (Fig. 3). Combined treatment with gemcitabine and probiotics also led to lower vimentin and Ki-67 staining (Fig. 3). To understand if this outcome was related to the proportion of probiotic used, GMNL-133: GMNL-89 was used at a ratio of 1:1 with the simultaneous use of gemcitabine (200 mg/kg). However, pancreas histology revealed that the progression of PanIN lesions become more severe compared with when GMNL-133: GMNL-89 was administered in a 1:1/10 ratio (Fig. 4). When we used a 1:1 ratio of probiotics (GMNL-133: GMNL-89) but the oral gavage dosage increased to 10 times, the pancreas histology or vimentin and Ki-67 expression showed the lowest grade PanIN lesions in comparison with the previous results (Fig. 4). To study the effect of using probiotics alone (without gemcitabine), KC transgenic mice were treated with caerulein, then orally administered one-third (n = 3) or 10 times the dose (n = 3) of the original probiotic (GMNL133: GMNL89 = 1:1/10). Pancreatic sections revealed that high-dose probiotics without gemcitabine still had an inhibitory effect on PanIN lesions (Fig. 5).

### Blood biochemistry and blood cell counts

Biochemical analysis demonstrated the KC transgenic mice were prone to increases in their AST/ALT level after treatment with caerulein (25 μg/kg) and various dosages of gemcitabine (20, 50, or 100 mg/kg). We observed that the mice who received combined probiotics (GMNL133: GMNL89 = 1:1/10) generally had a lower AST/ALT level (Table 1) compared with the mice who did not receive probiotics. Table 2 shows that KC transgenic mice that were treated with caerulein (25 μg/kg) and a combination of gemcitabine (200 mg/kg) and 10 times higher doses of probiotics (GMNL133: GMNL89 = 1:1, 10x) had a significantly lower ALT level compared with the regular dose group (GMNL133: GMNL89 = 1:1). Even in the absence of gemcitabine treatment, caerulein-treated KC transgenic mice that received the high-dose probiotic (GMNL133: GMNL89 = 1:1/10, 10x) still generally showed a lower AST/ALT level in comparison with the low-dose probiotic (GMNL133: GMNL89 = 1:1/10, 1/3x) group (Table 3).

**Table 1:** Comparison of blood cell counts and biochemical values in KC transgenic mice after treatment with caerulein (Cae) and various doses of gemcitabine (GCB) with or without probiotics.

	Non-treated	Cae-GCB	Cae-GCB + Prob (1:1/10)
	KC mice	KC mice	KC mice
**GCB 20 mg/kg**	n = 3	n = 3	n = 3
RBC (M/uL)	9.46 ± 1.18	8.34 ± 0.09	8.76 ± 0.92
WBC (K/uL)	7.79 ± 1.72	4.60 ± 2.22	3.79 ± 2.05
PLT (K/uL)	808.00 ± 232.50	1184.50 ± 180.31	1231.00 ± 290.22
AST (U/L)	62.67 ± 10.02	114.00 ± 104.80	97.00 ± 65.51
ALT (U/L)	20.00 ± 6.08	72.33 ± 91.51	31.33 ± 26.86
Glucose (mg/dL)	322.00 ± 45.40	206.67 ± 23.86	125.33 ± 12.22^a^
BUN (mg/dL)	26.07 ± 1.05	41.00 ± 13.98	38.77 ± 6.41
Cr (mg/dL)	0.22 ± 0.03	0.27 ± 0.12	0.22 ± 0.02
**GCB 50 mg/kg**	n = 3	n = 1	n = 2
RBC (M/uL)	9.46 ± 1.18	8.50*	7.68 ± 0.47
WBC (K/uL)	7.79 ± 1.72	3.90*	6.06 ± 1.49
PLT (K/uL)	808.00 ± 232.50	1104*	1417.50 ± 340.12^d^
AST (U/L)	62.67 ± 10.02	87.00*	101.33 ± 61.60
ALT (U/L)	20.00 ± 6.08	30.00*	23.00 ± 5.00
Glucose (mg/dL)	322.00 ± 45.40	261.00*	68.33 ± 18.04^a^
BUN (mg/dL)	26.07 ± 1.05	29.20*	33.13 ± 2.01^a^
Cr (mg/dL)	0.22 ± 0.03	0.23*	0.22 ± 0.01
**GCB 100 mg/kg**	n = 3	n = 2	n = 3
RBC (M/uL)	9.46 ± 1.18	8.16*	7.65 ± 0.69
WBC (K/uL)	7.79 ± 1.72	4.63*	4.47 ± 0.23^b^
PLT (K/uL)	808.00 ± 232.50	979.00*	1136.00 ± 42.43
AST (U/L)	62.67 ± 10.02	156.00 ± 49.50	62.00 ± 8.89^a^
ALT (U/L)	20.00 ± 6.08	46.50 ± 20.51	18.00 ± 2.00^a^
Glucose (mg/dL)	322.00 ± 45.40	221.00 ± 11.31	159.00 ± 16.82^a^
BUN (mg/dL)	26.07 ± 1.05	29.70 ± 4.67	42.23 ± 1.51^a^
Cr (mg/dL)	0.22 ± 0.03	0.24 ± 0.06	0.25 ± 0.05

*ALT* alanine aminotransferase,* AST* aspartate aminotransferase,* BUN* blood urea nitrogen,* Cae-GCB* caerulein(25 μg/kg) + gemcitabine; Cae-GCB + Prob (1:1/10), caerulein (25 μg/kg) + gemcitabine + probiotics (GMNL133: GMNL89 = 1:1/10); Cr, creatinine; KC mice, *LSL-Kras*^*G12D/−*^-Pdx-1-Cre transgenic mice; PLT, platelet; Prob, probiotics; RBC, red blood cell; WBC, white blood cell.

Data are presented as mean $\pm$ standard deviation (SD).

^a^ *P* ≦ 0.05 differences between Cae-GCB and Cae-GCB + Prob (1:1/10).

^b^ *P*≦ 0.05 differences between Non-treated and Cae-GCB + Prob (1:1/10).

* n = 1.

**Table 2:** Comparison of biochemical values in KC transgenic mice after treatment with caerulein (Cae) and gemcitabine (GCB) with different doses of probiotics.

	Non-treated	Cae-GCB 200 + Prob (1:1)	Cae-GCB 200 + Prob (1:1) 10x
	KC mice	KC mice	KC mice
AST (U/L)	62.67 ± 10.02	222.75 ± 120.82	127.67 ± 80.15
ALT (U/L)	20.00 ± 6.08	59.75 ± 12.53	36.00 ± 21.17^a^
Glucose (mg/dL)	322.00 ± 45.40	196.75 ± 18.23	212.33 ± 38.11
BUN (mg/dL)	26.07 ± 1.05	32.30 ± 7.52	35.93 ± 20.34
Cr (mg/dL)	0.22 ± 0.03	0.20 ± 0.00	0.28 ± 0.11

ALT, alanine aminotransferase; AST, aspartate aminotransferase; BUN, blood urea nitrogen; Cae-GCB 200 + Prob (1:1), caerulein(25 μg/kg) + gemcitabine (200 mg/kg) + probiotics(GMNL133: GMNL89 = 1:1); Cae-GCB 200 + Prob (1:1) 10x, caerulein (25 μg/kg) + gemcitabine (200 mg/kg) + probiotics (GMNL133:GMNL89 = 1:1, 10 times); Cr, creatinine; KC mice , *LSL-Kras*^*G12D/−*^-Pdx-1-Cre transgenic mice; Prob, probiotics.

Data are presented as mean $\pm$ standard deviation (SD).

^a^ *P*≦0.05 differences between Cae-GCB 200 + Prob (1:1) and Cae-GCB 200 + Prob (1:1), 10x.

**Table 3:** Comparison of biochemical values in KC transgenic mice after treatment with caerulein (Cae) and different doses of probiotics.

	Non-treated	Cae + Prob (1:1/10) 1/3x	Cae + Prob (1:1/10) 10x
	KC mice	KC mice	KC mice
AST (U/L)	62.67 ± 10.02	199.67 ± 123.52	141.67 ± 58.48
ALT (U/L)	20.00 ± 6.08	73.67 ± 38.18^b^	47.33 ± 19.60
Glucose (mg/dL)	322.00 ± 45.40	212.33 ± 18.90	151.67 ± 106.88
BUN (mg/dL)	26.07 ± 1.05	33.87 ± 0.74	29.70 ± 4.31
Cr (mg/dL)	0.22 ± 0.03	0.26 ± 0.03	0.21 ± 0.01^a^

ALT, alanine aminotransferase; AST, aspartate aminotransferase; BUN, blood urea nitrogen; Cae + Prob (1:1/10) 1/3x, caerulein(25 μg/kg) + probiotics (GMNL133:GMNL89 = 1:1/10, 1/3 times); Cae + Prob (1:1/10) 10x, caerulein (25 μg/kg) + probiotics(GMNL133: GMNL89 = 1:1/10, 10 times); Cr, creatinine; KC mice, *LSL-Kras*^*G12D/−*^-Pdx-1-Cre transgenic mice; Prob, probiotics.

Data are presented as mean $\pm$ standard deviation (SD).

^a^*P*≦0.05 differences between Cae + Prob (1:1/10) 1/3 × and Cae- + Prob (1:1/10) 10x.

^b^*P*≦0.05 differences between Non-treated and Cae + Prob (1:1/10) 1/3x.

## Discussion

In the past two decades, research into inflammation and the pathogenesis of cancer has demonstrated the tumor-promoting effect of immune cells (mainly the innate immune system) on tumor development. Studies have shown that inflammation is associated with multiple stages of carcinogenesis and can supply bioactive molecules to the inflammatory tumor microenvironment, including growth factors, survival factors, proangiogenic factors, induction signals for EMT, and extracellular matrix-modifying enzymes, which are known to promote cancer invasion and metastasis^19^. Conversely, we now recognize that the immune system also plays a crucial role in host immune surveillance, which inhibits carcinogenesis by identifying and destroying nascent transformed cells^20^. It was generally considered that the dual roles of inflammatory cytokines were associated with cancer suppression and progression^21^. PDAC is the most common and often lethal form of pancreatic cancer, and it is also considered to be typical of an inflammation-driven cancer^22^. As mentioned above, controlling or reversing the cancer inflammatory microenvironment is likely to be one feasible approach for pancreatic cancer treatment. In both animal experiments and human trials, probiotics produce anti-inflammatory metabolites and have been proven to actively facilitate resolution of inflammation in various inflammatory and autoimmune diseases, including ulcerative colitis, rheumatoid arthritis, systemic lupus erythematosus and multiple sclerosis^23^. Research studies have confirmed that the progression of pancreatic cancer is directly related to acute pancreatitis and chronic pancreatitis^24^. To clarify the potential role of probiotics in the treatment of pancreatic cancer, we conducted this animal trial using *Kras*-*driven* GEMM of PDAC.

Interactions between intestinal cells and gut microorganisms play a crucial role in maintaining host health. In recent years, the potential anti-cancer properties of various probiotics have been evaluated in gastrointestinal neoplasms such as gastric cancer, colorectal cancer and liver cancer^25^. Several potential mechanisms involved in the anti-carcinogenic effects of probiotics have been suggested, including changes in the intestinal microbiota, immunomodulation, reduction of inflammation, inhibitory effects on carcinogenesis, improvement of nutrient absorption, production of antitumorigenic compounds, production of short chain fatty acids and inhibition of pathogen replication^23^. Accumulating evidence indicates that gut microbial dysbiosis is associated with the pathogenesis of acute and chronic pancreatitis^26^. Microcirculatory alterations, splanchnic vasoconstriction and ischemia–reperfusion damage contribute to intestinal damage and increase the permeability of the intestinal barrier, which facilitates the translocation of bacteria and toxic compounds from the gut to the blood during acute pancreatitis. This exacerbates inflammation of the pancreas and can subsequently lead to fibrosis or necrosis. In addition, microbes contributing to carcinogenesis of PDAC and modulating tumor response to therapy^27^. For PDAC, a recent study shows that human intestinal microbiome represents about 25% of the intratumoral microbiome, but is absent from the adjacent normal tissue. They also observe that the levels of CD8 + T cells and activated CD8 + T cells have significantly increased in tumors from mice received fecal microbial transplantation from long-term survivors^28^. Sethi et al. demonstrated that Th1 (IFNγ^+^CD4^+^CD3^+^) and Tc1(IFNγ^+^CD8^+^CD3^+^) cells in the TME were increased, and pro-tumor IL17a (IL17a^+^CD3^+^) and IL10 (IL10^+^CD4^+^CD3^+^) were decreased after gut microbiome depletion by oral antibiotics^29^. These findings suggest that there is microbial cross-talk between gut microbiome and PDAC microbiome, and influences the host immune response and tumor progression. Direct manipulation of the intestinal microbiota may be a potential strategy for PDAC treatment. A meta-analysis of six randomized controlled trials indicated that there is insufficient evidence to recommend routine use of probiotics in patients with severe acute pancreatitis^30^. However, various probiotic strains and therapeutic dosages are likely to present heterogeneous conclusions. In contrast, the findings of the current study provide supportive evidence that four weeks of *Lactobacillus paracasei* GMNL-133 and *Lactobacillus reuteri* GMNL-89 treatment are able to inhibit PDAC progression in mice regardless of whether they are used as a part of a combination therapy or as a high dose-monotherapy. However, this dose–response relationship is insufficient to extrapolate because so far only limited evidence has suggested that more probiotic bacteria yield a greater benefit^31^. It is worth including additional higher doses as endpoints in future studies.

*Lactobacillus paracasei* are gram-positive, facultatively heterofermentative lactic acid bacteria. They are found in normal human and animal intestinal flora and are utilized in dairy product fermentation or as a probiotic supplement^32^. *Lactobacillus paracasei* GMNL-133 was chosen for this trial because it has been proven to be effective in children with asthma (Th2-driven diseases). It has been shown to inhibit Th2 cytokine production and modulate the Th1/Th2 immune balance by increasing IFN-γ levels^33^. Previous research has shown that Th2 responses exhibit tumor-promoting effects in PDAC. The Th2 immune phenotype can be reversed into the pre-existing Th1 immune phenotype through the induction of IFN-γ, interleukin (IL)-12, or IL-27, which subsequently causes tumor-suppressive effects^34,35^. Konduri et al*.* reported that adjuvant Th1 dendritic cell vaccination combined with standard gemcitabine chemotherapy can provide durable protection against PDAC^36^. Furthermore, IL-6 is known to have pro-tumorigenic activity in the progression of PDAC^37^. Previous studies have shown that high serum IL-6 levels are linked with PDAC and are associated with a worse prognosis. In animal studies, the use of IL-6 signaling inhibitors reduced tumor growth in PDAC^37^. Supplementation with *Lactobacillus reuteri* GMNL-89, the second probiotic used in the current study, can cause antioxidant activity and reduced IL-6 levels in animal models^38^. Furthermore, probiotic strains that exhibit potential anti-carcinogenic properties may directly bind with mutagenic heterocyclic amines (HCAs), thus reducing intestinal HCA absorption, and eliminating HCAs through fecal residues^39–41^. Some studies support the theory that multi-strain probiotics are more beneficial than single-strain probiotics^42^. In the current study we increased the proportion of GMNL-89 probiotic administered but there was no beneficial effect. From this we speculate that the intake of higher doses of GMNL-133 probiotics may be more helpful in improving PDAC. This study supports the theory that high doses of probiotics produce better inhibition of cancer progression. However, there is currently insufficient evidence to support the additive or synergistic effects between these two probiotics in vitro for PDAC.

Gemcitabine is a chemotherapy agent widely used to treat various types of cancer, including non-small cell lung cancer, bladder cancer, breast cancer, and pancreatic cancer^43^. Accumulating evidence has shown that the therapeutic tolerability of gemcitabine is highly dose schedule dependent^44^. The most frequently reported side effects include bone marrow suppression, skin rash, altered liver function tests, flu-like syndromes and fever^45^. Following treatment with gemcitabine combined with probiotics, we observed a notable change in liver enzymes. Gemcitabine caused abnormalities in liver function tests for AST and ALT were reported in 67% and 68%, of cases, respectively^46^. In the present study, a general elevation in liver enzymes was noted after treatment with gemcitabine. However, low levels of liver enzymes were noted following the use of a combination probiotic therapy, particularly when a high-dose probiotic treatment was used. High-dose probiotics alone not only inhibited the progression of PDAC but also had a beneficial effect on liver enzyme levels. In addition, a slight decrease in white blood cells and an increase in platelet levels were observed in mice receiving gemcitabine treatment in the present study. Although thrombocytopenia is a well-known side effect of gemcitabine, thrombocytosis has also been reported^46^.

This study provides some important implications for the care of pancreatic cancer patients. The effects of probiotics are strain-specific, and the same probiotic in different doses may exert different modulating effects. As the effect of traditional chemotherapy is still not satisfactory despite decades of use, the addition of probiotics as an adjuvant or combination therapy should be considered as a potential treatment method. However, there were several limitations to this study. First, although GEMM PDAC is remarkably similar to human PDAC, the etiology is not exactly the same. Human PDAC commonly occurs in elderly patients and develops as a single neoplastic focus, but most of the tumors in GEMM are multi-focal^47^. Second, given the complexity of human tumor development, the mouse model may be too simplified. Third, cross-talk between host and gut microbiota is regarded as host-specific, thus the effects seen in the mouse model may not be observed in humans. Finally, disparity in experimental results may occur in different mouse models. Randomized control trials in humans are needed to determine the role of probiotics in cancer treatment.

In conclusion, the current study suggests that probiotics together with chemotherapy could help decrease the side effects of chemotherapy and simultaneously achieve better treatment outcomes.

## Data Availability

The data that support the findings of this study are available on request from the corresponding author. There are restrictions to the availability of mice due to material transfer agreement.

## Abbreviations

ALT: Alanine aminotransferase
AST: Aspartate aminotransferase
EMT: Epithelial to mesenchymal transition
GEMMs: Genetically engineered mouse models
HCAs: Heterocyclic amines
H&E: Hematoxylin and eosin
PanIN: Pancreatic intraepithelial neoplasia
PDAC: Pancreatic ductal adenocarcinoma
